# Guava borer worm (Lepidoptera: Cossidae), a limiting pest in guava: biology, lifecycle and management alternatives

**DOI:** 10.1016/j.heliyon.2019.e01252

**Published:** 2019-02-22

**Authors:** Víctor Camilo Pulido Blanco, Orlando Ildefonso Insuasty Burbano, Zaida Xiomara Sarmiento-Naizaque, Julio Ramírez Durán

**Affiliations:** a Facultad de Ciencias, Universidad Pedagógica y Tecnológica de Colombia, Corporación Colombiana de Investigación Agropecuaria (Agrosavia), Centro de Investigación Tibaitatá, sede Cimpa, km 2 antigua vía a Cite, Barbosa, Santander, 684511, Colombia; b Corporación Colombiana de Investigación Agropecuaria (Agrosavia), Centro de Investigación Tibaitatá, sede Cimpa, km 2 antigua vía a Cite, Barbosa, Santander, 684511, Colombia

**Keywords:** Agriculture, Environmental science

## Abstract

In 2006, a new limiting pest in guava was reported in the *provincia de Vélez*, Santander, Colombia, known as the borer worm. There is no knowledge of the biology of this pest so far. The aim of this study, conducted between May 2013 and December 2014, was to establish the taxonomy, lifecycle, damage (distribution, incidence, and severity), and control alternatives of this new limiting pest in guava, as input for an integrated management program. Results showed that this pest corresponds to *Simplicivalva ampliophilobia* (Davis et al., 2008). The life cycle in the field lasts 330–360 days, with one generation per year (univoltine): egg, 15–30 days; larva, 270 days; prepupa, 15 days; pupa, 30 days; adult, 15–30 days. Under laboratory conditions the lifecycle lasts 259–266 days, unknown egg state duration; larva, 7–8 instars, 210 days; pupa, 42 days; imago, 7–14 days (*n* = 60, α = 5%, 25.4 °C ± 4.93 °C, 55.6% ± 11.58% RH, photoperiod 0: 24). The incidence was 94% in 124 silvopastoral system farms with 7.51 ± 1.69 infested trees compared to 40.74 ± 5.52 observed trees (*n* = 4,970). The technified system showed a reduction in the average incidence of 50.83% compared to the silvopastoral guava cropping system. Severity was moderate (*n* = 48) in both systems. Morphometric descriptions of eggs, larvae, pupae, and imagos were performed. Damage and associated symptoms occur when larvae remove the conducting tissues of the plant adjacent to the medulla. Crop technification combined with the use of the parasitoid *Apanteles* sp., and the fungi *Metarhizium anisopliae* and *Lecanicillium lecanii* represent an alternative control for this pest.

## 1 Introduction

Colombia is the eighth largest guava (*Psidium guajava* L., 1753) producing country worldwide (Morales et al., 2010; Agenda de Innovación Estatal Guerrero, 2012), and at the national level, it represents the fourth largest fruit crop with 16,124 cultivated hectares. Moreover, it shows production of 239,713 t/year, and an internal rate of return of 46.39 (Carrillo and Ocampo, 2011). Guava is mostly cultivated in farming units smaller than 2 ha in scattered silvopastoral systems that are found at altitudes from the sea level up to 1,900 m a.s.l. (Morales et al., 2010). However, the participation in the national GDP is negligible due to serious phytosanitary problems and the marked technological gap it undergoes (Insuasty et al., 2007). These situations threatens to hinder the 9% annual growth this fruit is showing (Carrillo and Ocampo, 2011), exacerbating the difficult socioeconomic conditions of producers and collectors.

The *provincia de Vélez* in the department of Santander (PV) and the *Hoya del Río Suárez* (HRS) between the departments of Santander and Boyacá contributes with 33% of the national guava production (Cadena Agroalimentaria de la Guayaba y su Industria CAGI, 2013). This production is mostly destined to the “bocadillo” agroindustry (sweet snacks elaborated from guava that are extremely popular throughout the country) whose annual profits are around $ US 24,000,000 (CAGI, 2013) distributed among 131 factories that employs the labor of 6,000 families in the region (Insuasty et al., 2007).

Within the pest complex of guava cultivation in this region the fruit flies *Anastrepha striata* (Schiner, 1868) and *Anastrepha fraterculus* (Wiedemann, 1830) (Diptera: Tephritidae), as well as the guava weevil *Conotrachelus psidii* (Marshall, 1922) (Coleoptera: Curculionidae) (Leiva, 2012), are the most significant. In addition to these pests, the stem borer known as “*gusano taladrador*” in Spanish or "borer worm" is now included in the abovementioned list.

The borer worm was described for the first time by guava producers of the department of Santander, Colombia in 2006, and this new pest is now considered as one of the most important that affects significantly guava cultivation (Pulido, 2013, 2014). Furthermore, this author states that currently, this new pest causes substantial tree death with subsequent loss of productive units in this agricultural sector (Pulido, 2013).

**Fig. 1:**
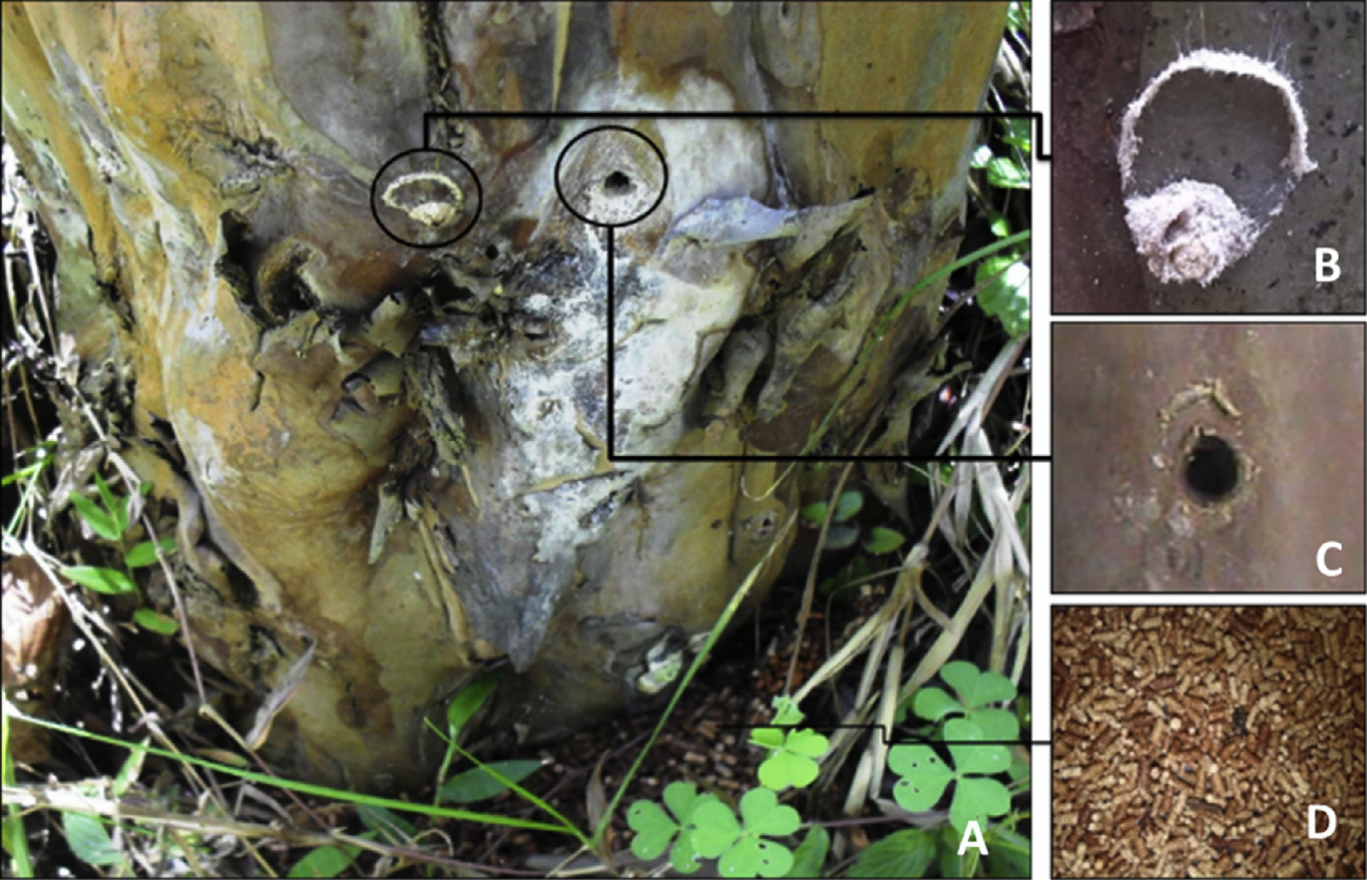
External appearance of the habit of the borer worm. A. Guava stem with two boring holes outlined. B. Capped hole (with operculum). C. External view of the excretory orifice without operculum. D. Borer worm excreta accumulated throughout the year at the base of the tree.

The only information that is available regarding the larval stage of this borer worm is that it feeds by piercing the guava tree stem with a fiber operculum in an orifice, from which separate wooden cylinders of different sizes are eliminated, the excreta, that accumulate at the base of the stem (Fig. 1) (Pulido, 2013). The other aspects of its biology, such as life cycle, distribution and extent of damage were unknown.

Regarding this new pest and according of the aforementioned, the aim of this study was to establish all the aspects of its biology, including its taxonomic identification, stages and duration of its lifecycle, distribution, severity, and damage incidence, as well as the identification of potential control alternatives that nurtures an integrated management program in commercial guava fields in the departments of Santander and Boyacá, Colombia.

## 2 Materials and methods

### 2.1 Study site

The experimental work was carried out in the field in plots located in various regions as follows. In the *provincia de Vélez*, department of Santander, Colombia, in the municipalities of Vélez, Jesús María, Guavatá, Puente Nacional, San Benito, and Barbosa. Additional plots were located in the municipality of Moniquirá in the *provincia de Ricaurte* in the department of Boyacá, and the municipalities of Briceño and Tununguá in the western region of the same department. Laboratory assessments were carried out in the agricultural entomology laboratory of C.I. Tibaitatá - Cimpa ascribed headquarters to Corporación Colombiana de Investigación Agropecuaria (Agrosavia), located at 5° 56′ 51″ N and -73° 36′ 24″ W.

### 2.2 Taxonomic identification

Descriptions of borer worm imagos and subsequent taxonomic investigations of the possible genera were corroborated with national and international specialists. The taxonomic species was established consulting the specialist Steve R. Davis, Ph.D. in Entomology, Department of Ecology and Evolutionary Biology at the Smithsonian National Museum of Natural History, and professor at the University of Kansas, through the *ex situ* examination of the stages of the pest, including three male and three female individuals.

Moreover, to identify the family of the pest, the lepidopteran code of the Taxonomy Research and Information Network of the Government of Australia was used in its online version (CSIRO, 2013). The material identified was preserved in the insectary of the agricultural entomology laboratory of C.I. Tibaitatá - Cimpa. Morphometric descriptions of the biological stages were carried out with the support of specialized literature and employing scanning electron microscopy following the method published by Bozzola and Russel (1992). For this, samples were fixed in a mixture of 5% glutaraldehyde and paraformaldehyde at 4% in 80 mM of cacodylate buffer and CaCl_2_ (5 mM) at 4 °C (Karnovsky, 1965). Subsequently, these were dehydrated with four ethanol solutions in ascending concentrations (50%, 60%, 70%, and 96%) for 15 min and placed in an intermediate solution of amyl acetate to perform a drying process of its critical point using CO_2_. A gold coating was applied in the exterior for 120 seconds and 140 mA under low-pressure conditions to achieve the conductivity required. After sample preparation, photographs were taken outlining the position of the main setae of the head, the thorax and the abdomen of the pest, and employing related studies for Cossidae.

### 2.3 Lifecycle

In the field, the biological stages of the insect were studied employing data obtained from weekly damage inspections, collection densities, and information of producers, according to the attack of the pest over time. Results were validated in the laboratory using population density corroborations according to the time of the year in commercial guava fields in the PV and in the HRS.

Adults were collected employing handmade traps in the emergence holes, consisting of plastic bottles and PVC tubes with one end open towards the trap and the other capped with tulle fabric; further, also red, green, yellow, blue and white pit-light traps, and Wilkinson and intersection traps (Peck and Davies, 1980).

For egg collection, a random tree was chosen from each experimental farm with recent damage presence due to borer worm larvae, and whose base had not been trafficked. Further, a soil sample (140 g ± 6 g/tree) of this area was collected once a week, in three trees per farm, sampling four farms and 6 replicates. Leaves, roots, and stones were checked for borer worm eggs before being discarded so these would not damage the sieves. A successive screening was then carried out, beginning with sieve No. 6 (3.35 mm), No. 8 (2.36 mm), No. 12 (1.00 mm), No. 16 (1.18 mm), No. 25 (0.710 mm), No. 30 (0.600 mm), and up to No. 200 (0.075 mm) (ASTM E-11 of 1995). The sifted material was observed at 40x with an SZ-PT OLYMPUS® stereoscope (1.7–40x). Larvae and pupae collection were made based on the presence of fresh and large excreta, and then the trunk was sectioned using a chainsaw, a hammer, and a metal wedge.

Under laboratory conditions, the lifecycle of the insect was studied from collections of biological samples of different pest stages (eggs, larvae, and pupae) carried out in farms of the productive guava sector, and selected based on the infestation results of 2013 subject to availability, and in the municipalities mentioned above. The specimens were studied emulating field conditions, i.e. temperature of 25 ± 3 °C, relative humidity of 60 ± 10% and light/darkness (larval photophobia) with a photoperiod of 0: 24. Eggs were kept in 60 mm Petri dishes on moistened filter paper. The data on morphology, color (Munsell and RGB color tables) and size (width and height) were obtained using a stereoscope and a microscope with a micrometric of 1: 100 mm at 4X and 10X. These were reviewed daily, registering the number of hatches and non-viable eggs. Dead eggs were kept in a glass container with a butyl cap and alcohol at 40% v/v, until completing 30 units. Then, these were labeled with the type, date and responsible person.

Once larvae were brought from the field they were measured with an entomological vernier in order to register and establish intervals by size. These intervals were established with a confidence level of 95% (α = 0.05, *n* = 40) as follows: small (from 15 mm to 1.5 cm), medium (from 2.0 cm to 4.0 cm), and large (from 4.5 cm onwards). Larvae were then cleaned inside a laminar flow cabin, including successive washes with 0.1% v/v solution of NaClO for 30 seconds and rinsed with distilled water for 1 minute. These were then separated into two groups: the first instar larvae were kept in transparent plastic cups with a square of 1 cm^3^ of the artificial diet published by García del Pino and Haro (1986) provided until they pupated, with 30 larvae in total. This diet was changed once every two weeks or until it was consumed completely. The second group of larvae was kept in a bioassay fed on different diets.

Larvae were observed daily in search of cephalic capsules to establish the number of larval stage instars. The distance between the gena, diameter, width, and height of the capsule was measured. The data obtained were analyzed employing Dyar's coefficient and was contrasted with the number of molds observed. Based on the estimated value of Dyar's coefficient and the larval stages, a linear regression analysis published by Parra and Haddad (1989) was carried out employing the statistical package R 3.5.0.

The diet of the last instar larvae was changed to a holidic diet consisting of fresh guava sawdust. The second group was subjected to a complete unifactorial and randomized design with four treatments and four replicates. A response unit comprised a borer worm larva in a centrifuge tube containing 20 cm^3^ of diet and three larvae per experimental unit (two medium-sized and one large-sized), for a total of 52 larvae. The diets tested included four diets as follows: C (Control); Zp (semi-synthetic diet published by García del Pino and Haro (1986) for *Zeuzera pyrina* (L., 1761) (Lepidoptera: Cossidae) modified with guava sawdust); CL (semi-synthetic diet established by Iglesias et al. (1989) for lignicolous Coleoptera, modified with guava sawdust); and Hg (semi-synthetic diet mentioned by Vargas et al. (2001) for *Hypsipyla grandella* (Zeller, 1848) (Lepidoptera: Pyralidae) modified with guava sawdust and chloramphenicol). Diets are detailed further in Table 1. Containers were labeled with a consecutive number that corresponded to the randomization code assigned to each experimental unit, foisted to a treatment and a replica.

**Table 1:** Diets evaluated for the maintenance and development of the borer worm larvae under laboratory conditions.

Ingredients	C (control)	Zp (García del Pino and Haro, 1986)	CL (Iglesias et al., 1989)	Hg (Vargas et al., 2001)
Guava sawdust (g)	20	190	44	2
Agar-Agar (g)	15	24	10	20
Sterile water (cm^3^)	1,000	1,100	200	850
Methyl-parahydroxybenzoate (g)	1.4	3	1	1
Ascorbic acid (g)		7	0.6	
Benzoic acid (g)			1	
Linoleic acid (cm^3^)		3.4		
Sorbic acid (g)				2
Caseine (g)				20
Chloramphenicol (g)		0.25		0.3
Cholesterol (g)				0.2
Ethanol (cm^3^)			5	
Wheat germ (g)			44	120
Corn flour (g)			22	
Powder milk (g)		70		
Yeast (g)		70	11	15
A mixture of vitamins (g)				15
Sucrose (g)		40		30
Wesson salts (g)				10
Soya seeds (g)		100		

C (Control); Zp (semi-synthetic diet modified with guava sawdust); CL (semi-synthetic diet modified with guava sawdust); Hg (semi-synthetic diet modified with guava sawdust and chloramphenicol).

Pupae were kept on fresh guava sawdust. The dead ones were kept in a glass container with a butyl cap and alcohol at 70% v/v, until completing 30 units. They were labeled with their type, date and the responsible person. When pupae were found in narrow galleries and their direct extraction was risky for their survival, removal was carried out by cutting a portion of the guava tree of the same size as the container.

Adults were kept in entomological metal cages with walls of tulle fabric in a 2: 2 ratio including 6 pairs, a sprue, rehydrated guava flour, cotton impregnated with 10% sugar solution, another cotton impregnated with honey water at 10% and 2 g of multiflora pollen. Several paper napkins were placed on the walls of the cage in order to collect the postures. The number of eggs per posture was quantified, and the way in which eggs were laid as well as both the shape and size of the eggs were described. Dead imagoes were conserved in the pest insectary of the entomology laboratory of Cimpa, under the following standards: specimen fixed in an entomological needle 00, extended wings, labeled with its taxonomic identification, location, date, and collector. Several photographs of the specimens were sent to specialists for corroboration of their taxonomic identification.

### 2.4 Damage description

#### 2.4.1 Incidence

In the period between April 19 and November 1, 2013, random inspection visits were made to plots in areas cultivated with guava from the municipalities of Vélez, Barbosa, San Benito, Guavatá, Puente Nacional, and Jesús María in the department of Santander; and also to Moniquirá, Tununguá and Briceño in the department of Boyacá. Geographical positioning data of each locality, the percentage of trees affected, state of damage (fresh: active; outdated: inactive), type of host (*Psidium guajava* or *Myrtus communis*) and abiotic data (rainfall regime, temperature (° C) and relative humidity (RH in %)) were registered. In total 124 visits were carried out. The rural areas of the municipalities were chosen based on the presence of standing guava trees. In the field we choose farms at random tossing a coin.

#### 2.4.2 Severity

Four farms were selected as they showed the presence of the borer worm as well as another pest of guava (the “bander worm”) from a parallel study; additionally, weekly monitoring was agreed with the farm owners for a period from July 4, 2013, to December 24, 2014. In total, 72 trees were randomly evaluated located in four farms (two in the municipality of Moniquirá and two in Vélez); of these trees, 48 were of interest for the borer worm damage assessments, i.e. 12 individuals per farm were characterized (6 trees were infested with the borer worm, and 6 were healthy trees without the presence of this pests). Each tree was labeled with a consecutive number. Weekly inspections were carried to evaluate the injuries caused by the pests, registering presence, number and stage of the same (fresh: recent damage; outdated or old: inactive damage) as well as the symptomatology observed in each tree. All the lesions were marked with white ink to facilitate their monitoring and the description of their evolution. Data were registered based on three proposed severity scales with six categories defined for the pest (Table 2). The data and the results obtained from the rest of the 24 guava trees of the pest studied in parallel are not shown in this study.

**Table 2:** Proposed damage scale to monitor the severity of pests in commercial guava fields.

Degree	Symptom	Borer worm
0	Absent	Absent
1	Very mild	Outdated (old)
2	Mild	1 active
3	Moderate	2-4 active
4	Severe	5- 8 active
5	Very severe	≥9 active

At each inspection, temperature (maximum and minimum), relative humidity (%) and rainfall (mm) records were registered. Photographic records were also taken to help support symptomatology descriptions.

### 2.5 Management alternatives

#### 2.5.1 Cultural method

In the HRS there are two cultural management systems of guava production: the traditional system, in units of less than one hectare, with trees without density of sowing, without pruning of formation or fructification, in a state close to the sylvan. This system is called silvopastoral. The modern system, called technified, has management of the density of trees in several hectares, as well as the size, seasonality, fertilization and irrigation. In order to know the behavior of the pest, from the comparison of the two cultural systems of crop management, a correlational/causal transactional descriptive non-experimental design was used (ex post facto variables not deliberately manipulated). The answer was the absence/presence binomial of the borer worm. The causal correlation includes the binomial response compared to the system used in guava cultivation. The sample size was established using population density data of six locations and fixing these to a minimum area of 0.5 ha. A finite population sample size in a stratified sampling was calculated for each system (Table 3) with the above-mentioned data, using the following equation (Eq. 1):

(1) $$n=1+\frac{N.\;Z².\;p\;.\;q}{\left[\left(N-1\right)\;e²]\;+\;\left(Z^{2}.p.q\right)\right]}$$

**Table 3:** Sample size calculated for the cropping systems in the bioassay on incidence vs. cropping system.

Farm	Technified guava cropping system			Silvopastoral guava cropping system		
	Density	*n*	*N*	Density	*n*	*N*
A	222	69	194	18	9∗	140
B	200	62		158	80	
C	200	62		101	51	

∗ Total number of trees in one farm after pruning.

Where *n* = sample size; *N* = population size; *Z* = value from the normal distribution table with a confidence level of 95%; *p* = percentage of the population that has the desired attribute, in this case, the incidence of the borer worm *S. ampliophilobia*; *q = 1-p*; *e* = maximum estimated accepted error.

Likewise, for each stratum, proportionally (Eq. 2),

(2) ni=n*Ni/N

Where *ni* = sample size in each stratum, in this case, the experimental farms; *Ni* = population in each stratum, in this case, guava trees per hectare.

The exogenous microclimate and soil variables were homogenized by sampling agroecosystems of the same spatial location. In total, 194 trees were sampled in technified guava cropping systems and 140 in non-technified silvopastoral guava cropping systems during a period of four months. The presence of lesions caused by fresh, outdated and totally colonized by the pest insect was registered.

#### 2.5.2 Parasitoids

Parasitoids of eggs, larvae, and pupae naturally present in the study localities, and the ones collected in farms of the productive sector were identified. They were preserved in alcohol at 40% v/v for their taxonomic identification. The number of stages affected by each morphospecies was quantified, as well as the type of parasitism, the stage in which the pest was attacked, and the number of individuals of the parasitoid per attack. The most recurrent parasitoid was selected as an alternative control for the guava borer worm.

#### 2.5.3 Entomopathogenic fungi

These fungi that infect larvae and pupae present in samples brought from the field and those found in the laboratory were isolated and cultivated in Potato Dextrose Agar (PDA). The cultures were maintained at 28 °C and 80% RH for seven days, after which aerial mycelia samples were made in PDA microcultures under the same conditions. Once the macroscopic fruiting bodies were identified, direct observation was made in fresh at 40X using an Olympus BX40 fluorescence microscope for recognition of reproductive structures. Microcultures were identified by a phytopathology specialist from the biological control laboratory of the Faculty of Agronomy, U.P.T.C. of Tunja, Boyacá, Colombia.

## 3 Results and discussion

### 3.1 Taxonomic identification

The guava borer worm was identified as *Simplicivalva ampliophilobia* sp. nov. (Davis et al., 2008), a species recently reported for science in a study of the Cossulinae subfamily in Costa Rica. Disregarding genitalia studies due to the low number of specimens captured and obtained in the laboratory (*n* = 10), the diagnostic characters of the species were the net body size of the imago, the net body size of the larva, larval coloration patterns, coloration of the thoracic shield, size and pigmentation patterns of the termen, and shape, position and colors of disc stains. Relevant literature of the species with these characters reported for the Neotropics was sought, attaining descriptions published by Dognin (1916) for *Cossula nigripuncta*, a species originally described in Pacho, Cundinamarca, Colombia, at an altitude of 2,200 meters above sea level, as well as in Medina, Cundinamarca, at 500 meters above sea level (Dognin, 1916). However, the figures of the holotypes originally described were not found. The literature stated that *Biocellata,* belonging prior to the *Cossula* genus, was detached, described and established as a genus on its own. According to Davis et al. (2008), *Biocellata* is recognized by having two subterminal points (spots) in the front wing, a character found in the borer worm species under study. Finally, the description of *S. ampliophilobia* corresponded to the features shown by the borer worm samples, i.e. size, general color of the cephalic, thoracic and abdominal tagmas, venation, spots of the termen that run from the coast to the jute margin, color, shape and disposition of the pectinate antennae, all referred to in the holotype of a male specimen. The female imago, the pupa, and egg stages were unknown for this species and vague references to the larval stage were found. Therefore, descriptions of each stage have been elaborated in this study and are found as follows.

### 3.2 Egg

**Fig. 2:**
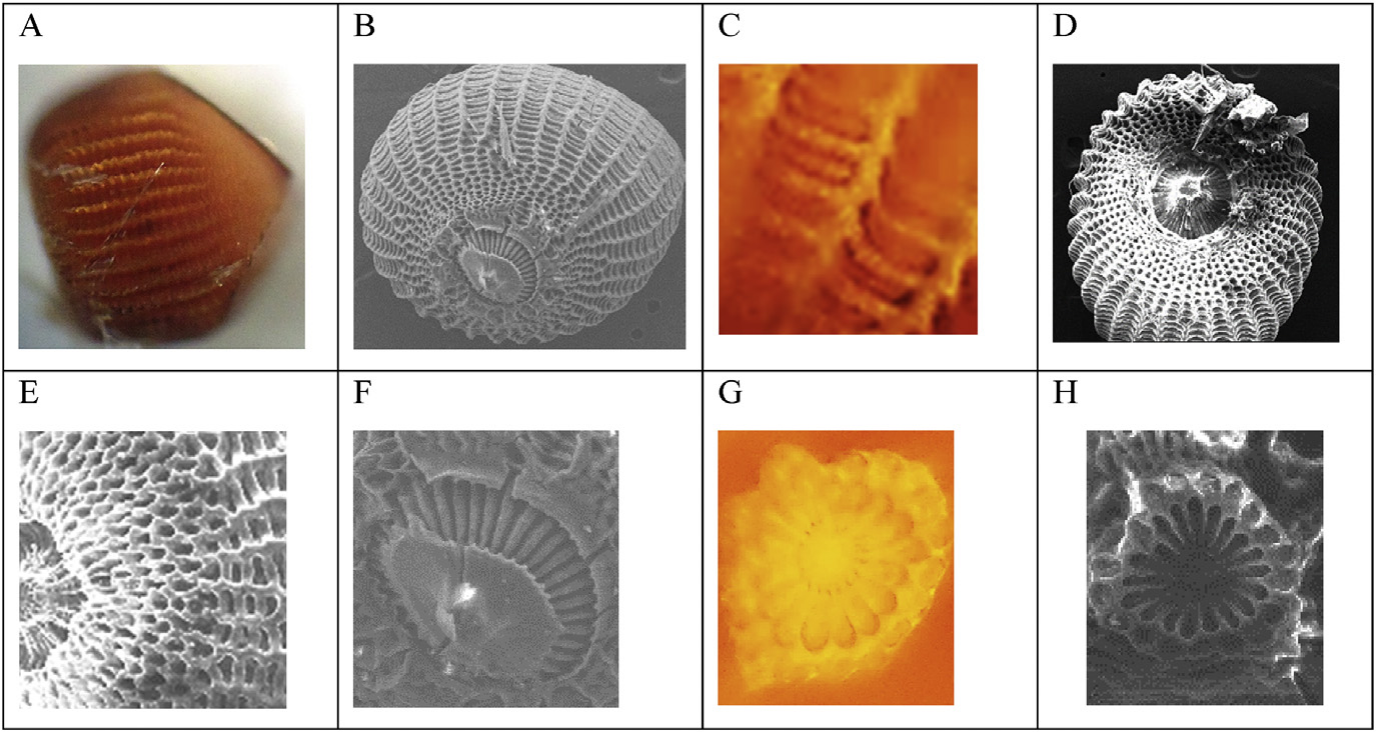
Eggs of *S. ampliophilobia*. A. Lateral view of the egg at 400 magnifications. B. Scanning microphotography of the lower pole of the egg with a detail of the radial coasts at 400 magnifications. C. Detail of the radial coast cells at 1000 magnifications. D, E. Scanning microphotographs of the micropylar rosette. F. Scanning microphotography of the lower pole with hexagonal cells. G. Detail of the micropylar rosette at 1000 magnifications. H. Scanning microphotograph of the micropylar rosette at 2000 magnifications. Source of microphotographs: Researcher Gloria Patricia Barrera, Agrosavia-UNAL, 2014.

(Fig. 2): from simple and spaced postures, on average 29 eggs were laid per posture (*n* = 4), the oviposition site is unknown, although it is suspected that it is cryptic just like egg coloring; hence, this is the reason why it was not possible to discover eggs in the field. Eggs obtained under laboratory conditions have a dark red color (2.5YR 3/6) in the Munsell® table or dark copper (# A33A00) in the RGB code. An egg measures in average 1.06 ± 0.086 mm in length and 0.9 ± 0.066 mm in width (α: 0.01; *n* = 20), it has a cylindrical to sub pyramidal shape (Fig. 2 A), with 38 radial coasts (*n* = 5), none of these in direct contact with the micropillary area of the anterior end of the egg (Fig. 2 B, C). The cells between the radial coasts and the aerophiles are rectangular with blunt ends (Fig. 2 D, E).

The anterior part of the egg gently curves until the invagination from where the micropylar rosette protrudes, without exceeding the height of the invagination curvature (Fig. 2 D, E). The cells of the previous part are hexagonal and occupy, on average, half the space of the coasts (Fig. 2 F). The rosette consists of 17 primary cells (*n* = 5) (Fig. 2 F). Its rear part is tapered with an acute abrupt angle from the zone where the coasts are attenuated (Fig. 2 G, H).

### 3.3 Larva

**Fig. 3:**
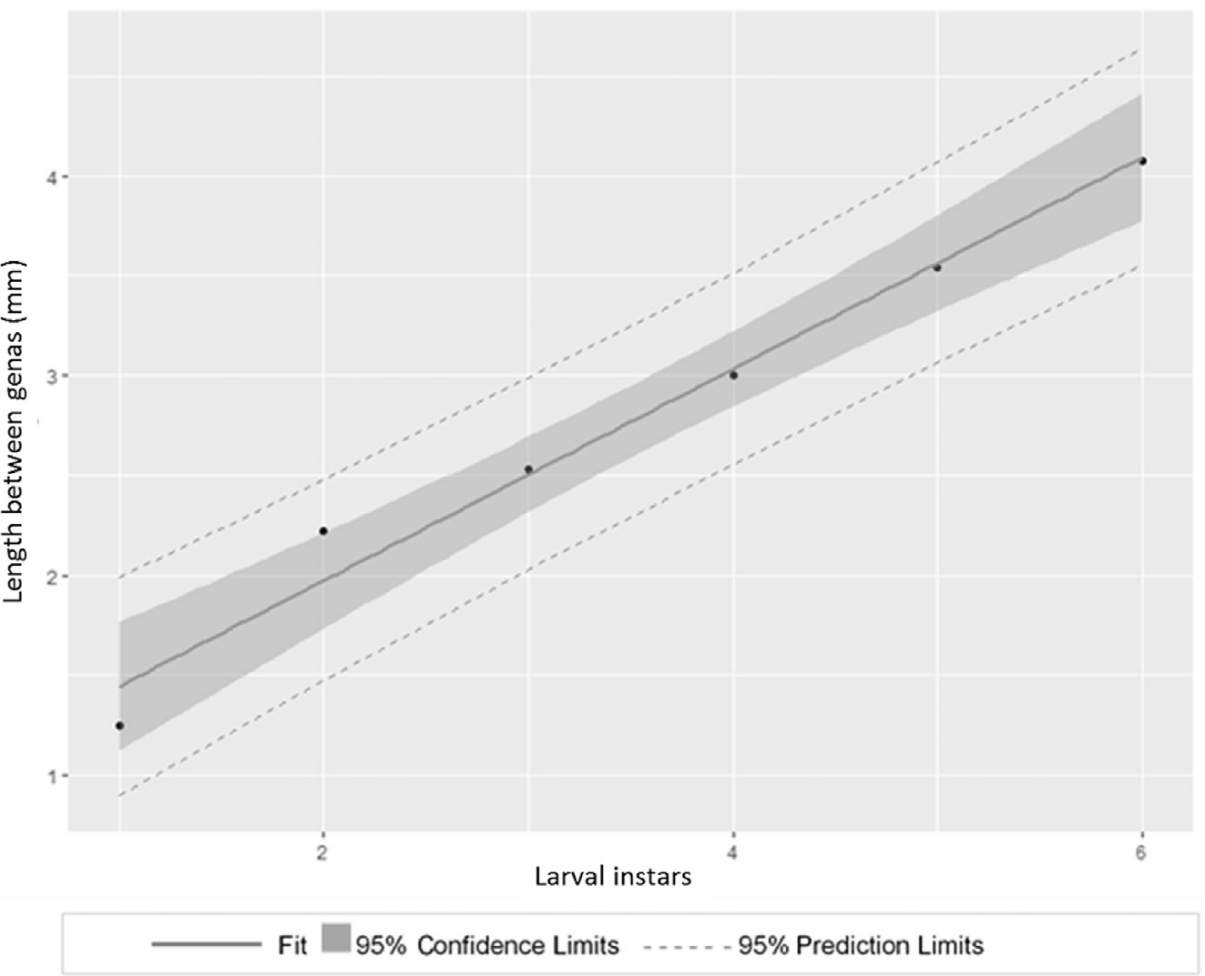
Relation among six instars and mean length between genas (mm) employing Dyar's coefficient (R^2^ adj = 0.9747, P < 0.05).

The estimation of Dyar's coefficient showed a direct relation of 98% between gena length values and six larvae stages under laboratory conditions (Fig. 3). In the first state, a high coefficient of variation (28.5) was found that could presume the omission of one or two initial states. Thus, *S. ampliophilobia* shows between 7 or 8 larval stages at 25 ± 3 °C, at a relative humidity 60 ± 10% and at a photoperiod of 0:24, fed with an artificial diet disclosed by García del Pino and Haro (1986). This polymorphic development has been widely reported in species that feed on seeds such as *Zeuzera pyrina* (García del Pino and Haro, 1987), and is due to environmental factors such as the temperature, the diet, and the vigor of the material used (Poitout and Cayrol, 1969).

**Fig. 4:**
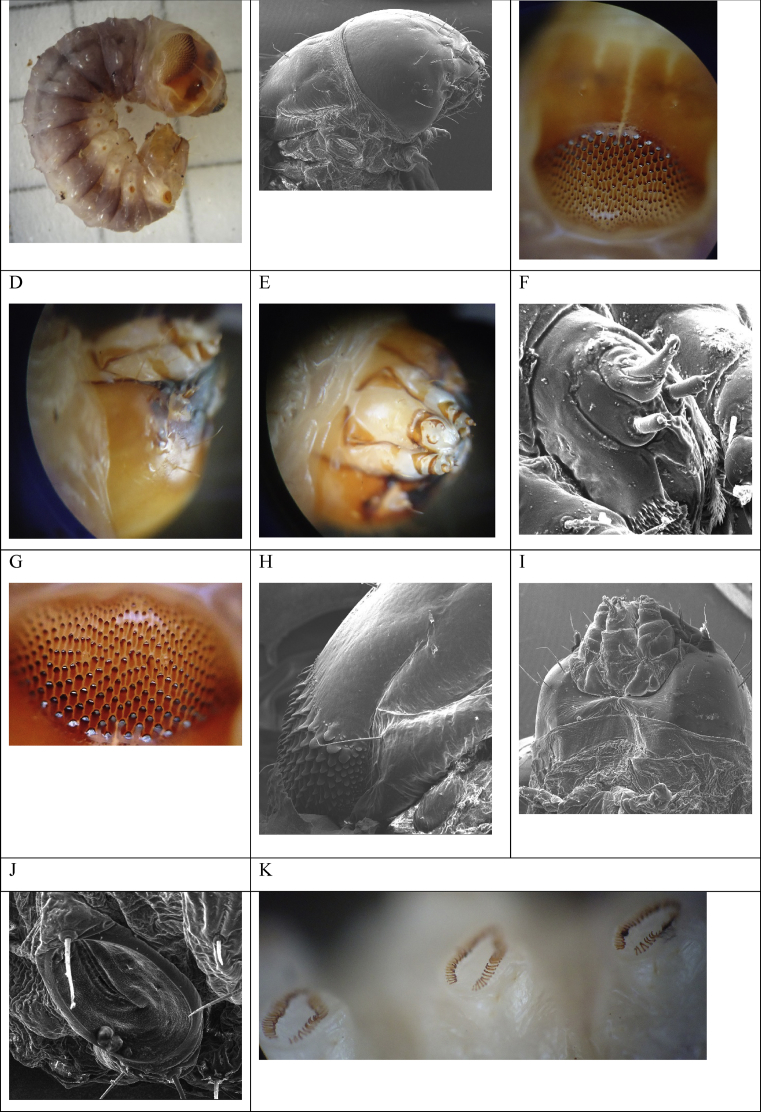
Larva of *S. ampliophilobia*. A. Top view of a last instar larva. B. Scanning microphotograph of the union of the cephalic and thoracic tagmas at 50X. C. Thoracic shield at 40X. D. Lateral view of the cephalic tagma at 10X. E. Bottom view of the cephalic tagma at 10X. F. Scanning microphotograph of the spinning apparatus at 200X G. View of the "drops" of the thoracic shield. H. Scanning microphotograph of the "drops" of the thoracic shield at 100X. I. Scanning microphotograph of the lower part of the cephalic tagma at 50X. J. Scanning microphotograph of the eighth abdominal spiracle at 200X. K. View of the prolegs with uniordinal crochets. Source of microphotographs: Researcher Gloria Patricia Barrera, Agrosavia- UNAL, 2014.

The eruciform larva of the last instar with a maximum registered size of 4.5 cm in length, has a grey color (5PB 6/) in the Munsell® table and also in the RGB code (#AAAAAA) on the back. The pleura color is gray and white with a nacre color (# FFFFF2) in the RGB code on the belly (Fig. 4 A). However, these features appear when it has been extracted and exposed to light. We have observed that the larva of the last instar is almost completely nacre inside the host, so its hypersensitivity to light could explain this condition, with subsequent photophobia in response to light stimulus. Furthermore, it has a strong enlarged head, with prominent genas slightly smaller than the pronotum (Fig. 4 B). The occiput flows with the vertex, brown on the sides and white at the center, with two lines that extend 2/3 of the length of these parts (Fig. 4 C). The frons is dark brown, and the sutures with the genas are darker and very conspicuous. The genas are white towards the upper central part, and brown on the sides and base (Fig. 4. D). The black mandibles are solid, short, robust with five teeth (Fig. 4 E). The brown antennae have only one antenite with a prolonged terminal keel, supported by a prominent gray scale and surrounded by a black circular suture (Fig. 4I). The thoracic shield is widened towards the head and compresses slightly towards the thorax. The ecdysial suture is conspicuous, and on both sides, there are two small, centric white lines. Below these lines, on each side, there is a dark brown patch with a half-moon shape. On the sides, there are two small brown sclerotizations (Fig. 4 C). Below the white lines, there is a strong pigmented brown area with very short projections. The most distinctive part of the thoracic shield is the posterior area: a convex curvature (shaped like a mountain) delimits a richly pigmented area, with a "drop" design (Fig. 4 G). Each "drop" runs with the thinnest and most diffused brown part from the anterior zone to a brown area concentrated towards the thorax (Fig. 4 G and H). As these move away from the curvature, the "drops" become smaller and less elongated. The spinning apparatus is composed of three spinners easily visible at 40X, two in the lower plane immediately next to the gena and a central one (Fig. 4 F, I). It has three pairs of thoracic legs and five spurs (propodia), four in segments 3A–6A and the last in segment 10A, basal (plesiomorphic), typical of Lepidoptera. Each thoracic tergum shows four processes on the sides: two small, anterior, with a very faint brown point, and two posterior, larger, and slightly displaced towards the center. In each pleura of the first thoracic segment, just in the area where the gray color of the tergum diffuses, there is a large brown circular stigma. Segments A1 to A7 have spiracles of the same brown color as the tegument of the vertex and are approximate of the same size. In Segment A8 a very large spiracle is observed, three and a half times larger than the previous ones and of equal size to that of the pronotum. This stigma is a diagnostic character of the Cossidae family (Fig. 4 A, J) (CSIRO, 2013), and is located just above the abdominal stigmata line. Crochets with transverse bands are uniordinal (Fig. 4 K). When the larva is disturbed, it secretes a red liquid through the mouth. Moreover, larvae excreta are cylinders three times longer than the widths of wood residues; these excretions accumulate at the base of the area where the damage is caused by the larva and is directly proportional to the size of the individual.

### 3.4 Pupa

**Fig. 5:**
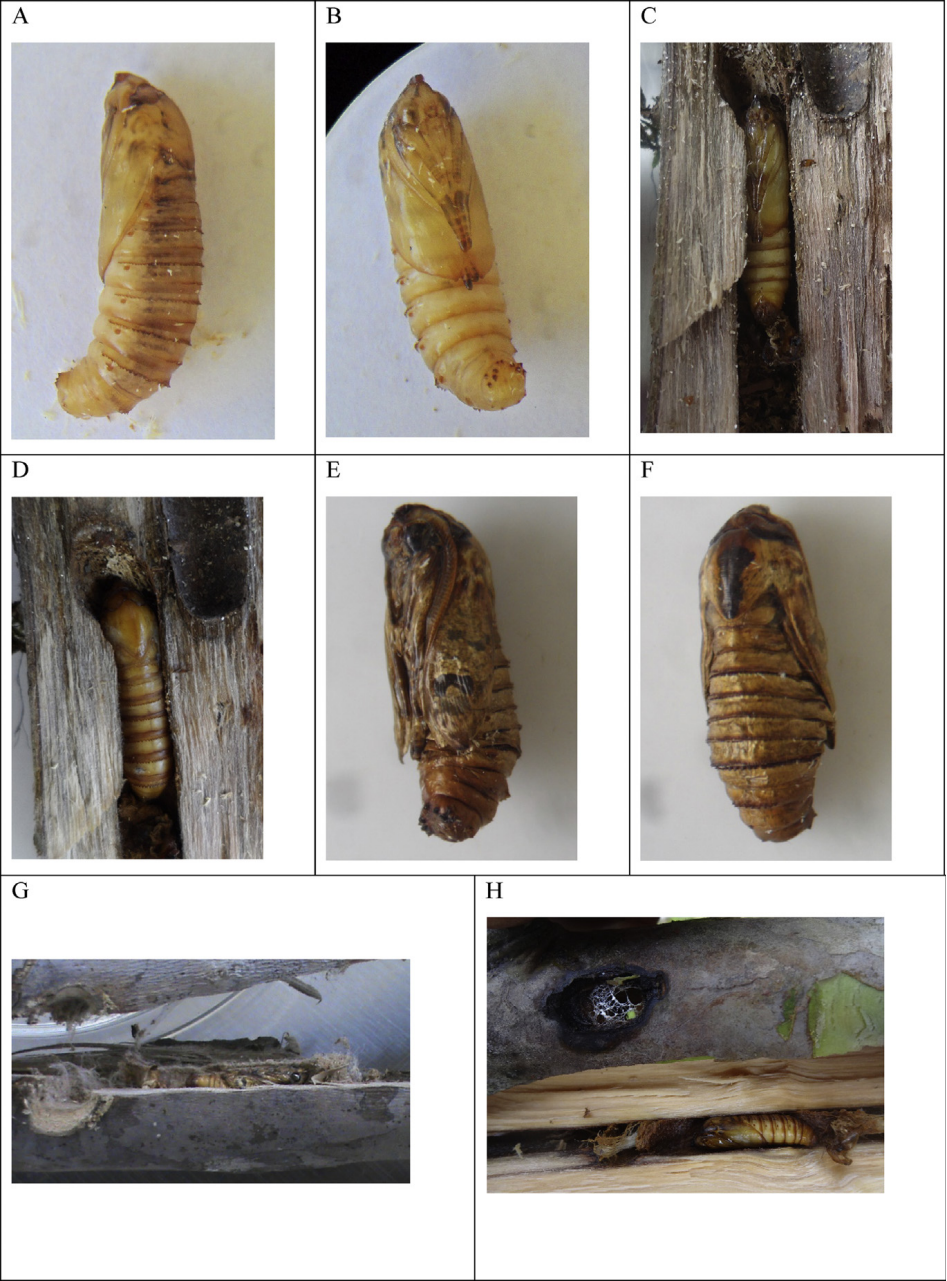

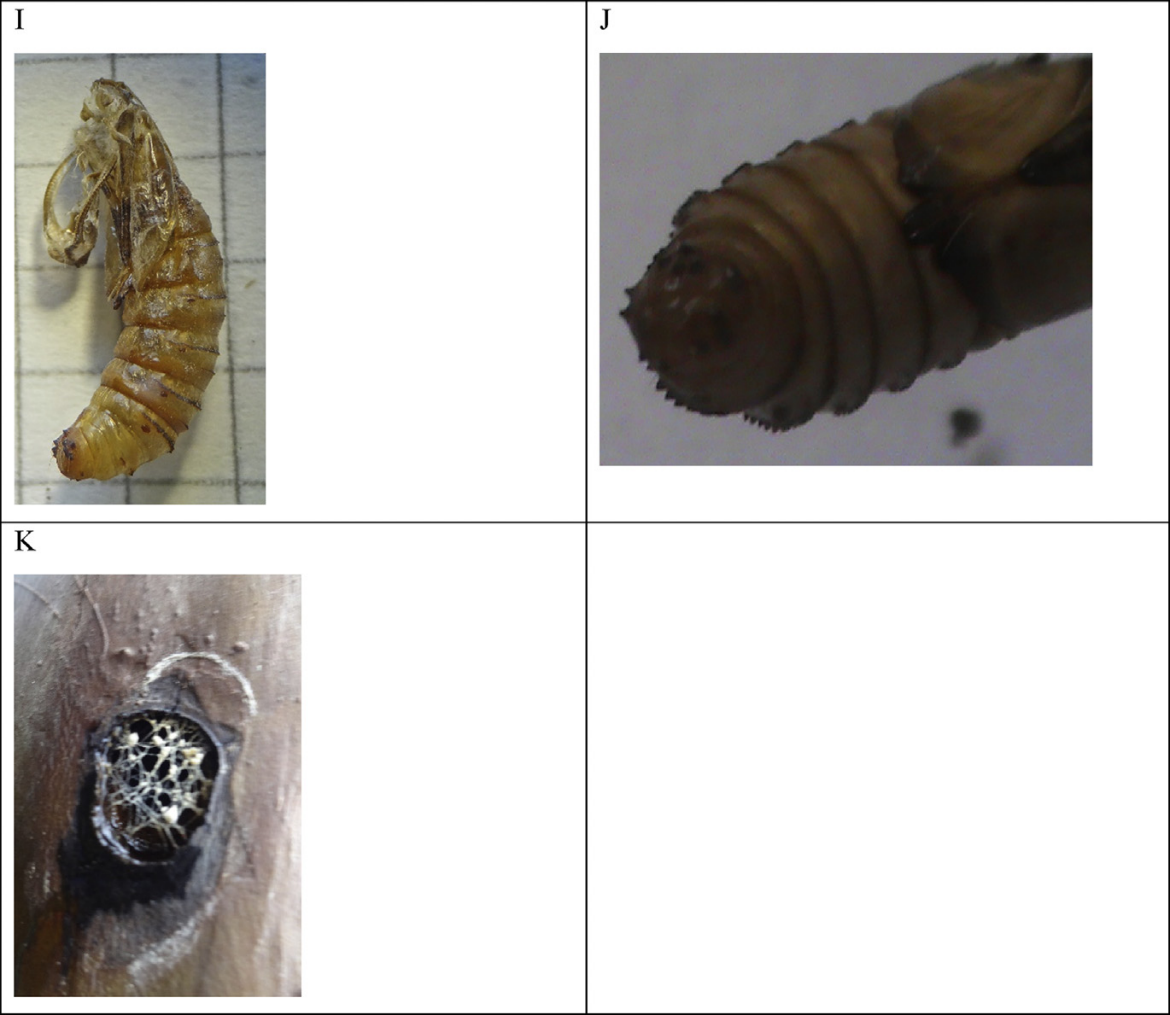
Pupa of *S. ampliophilobia*. A and B. Female pupa in the first stadium days. C and D. Female pupa in a guava branch. E and F. Male pupa in a stage close to adult emergence. G. Male pupa in a guava segment protected by a cocoon near the exit hole H. Female pupa in a guava segment protected by a cocoon near the exit hole. I. Exuvia. J. Detail of the apex of the pupa, spines, and spiracles. K. Diagnostic nature of pupae in the field.

Obtect, adectic, male pupae reach on average a size of 12.0 ± 0.3 mm in length and 5.0 ± 0.20 mm in width (*n* = 5); females pupae show an average size of 18.0 ± 0.35 mm in length and 4.5 ± 0.30 mm in width (*n* = 5). Pupae show a caramel color (5 and 7/8) in the Munsell® table and also in the RGB code (# FFAB00), with a smooth, shiny and translucent tegument, almost hyaline, where the growth and pigmentation of each tagma of the imago are distinguished. As the day of emergence approaches, several areas of the tegument become darker, e.g. the tarsus of the legs, the compound eyes, the occiput and the thorax (Fig. 5 A–F). Proboscis absent and labial palps are robust and long, thickened at the base, accompanied by two sinusoid sutures on the sides, below the compound eyes. Podothecas one and two with the base of the coxa very evident, curved and widened; podotheca one disappears below the alar chest and reappears in segment A3 (Fig. 5 A, C, and E). Terminal tarsi of the podothecas and apex of the alar chest strongly pigmented during the entire pupal development. Epicraneum pronounced with a large process, 4 mm long (Fig. 5 H). Frons with a concavity that forms an M in the inferior margin of the epicranial process. There is a long and hyaline ceratotheca where the pectens of the antennae are observed. Pronotum is curved with a high center, strongly pigmented in black. Diffused ecdysial sutures. Mesonotum shaped as a shield, with the black center elevated and the base sharpened. The insertion of the alar chest is conspicuous, as well as the main ecdial line. The mesonotum continues fluidly between the pronotum and the metathorax and covers the first abdominal segment.

The thorax does not show any process. Abdominal segments have a rectangular shape, elongated, with the base strongly pigmented with a caramel color. Segments A1 to A3 run below the alar chest without truncating it. All segments have a simple anterior-superior row of sclerotized spines; the spines do not reach the sternal zone. Abdominal segments 3 to 7 are mobile in males, 3 to 6 in females, T2 to 7 in males and 2 to 6 in females with two transverse rows of spines; T8 in males, T7 and T8 in females with a row of spines (CSIRO, 2013). There are elevated circular stigmas below the line of spines. Sternite of the same size as the tergum. Abdominal apex with sculptures shaped as sclerotized thorns, broad, thirteen in total, nine in the genital region and four in the anal region. Of the nine located in the genital region, eight are located on the sides, four in each side distributed in a square, and a central one. The four located in the anal region are separated into two groups of two on the sides of the centerline. The genital region is elevated and occupies two-thirds of the apex area of the pupa (Fig. 5 J). Cremaster absent. There is no evidence of the elaboration of a silk cocoon. A photograph of a pupa inside a guava trunk can be observed in Fig. 5 G and H. A diagnostic character of the presence of *S. ampliophilobia* pupa or prepupa in guava stems was found, i.e. presence of a silk reticule in the form of an imperfectly interlaced mesh (Fig. 5 K).

### 3.5 Imago

**Fig. 6:**
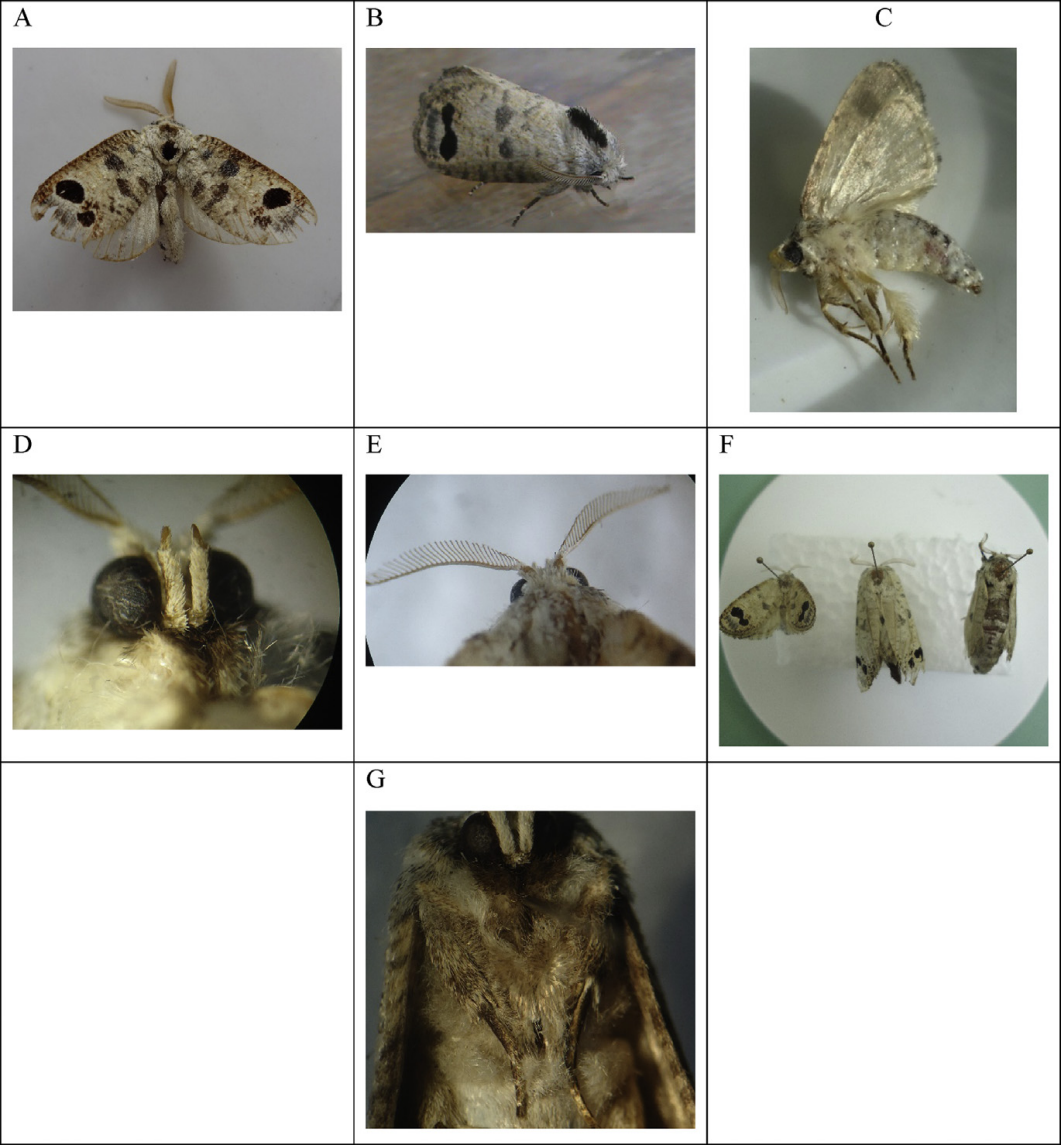
Imago of *S. ampliophilobia.* A. Male (view from above) B. Female (side view). C. Side view. Note the iridescence. D. Labial palps (absent glabella). E. Detail of the pectinate antennae. F. The difference in size between a female obtained in the laboratory (on the left) and two females captured in the field (on the right). G. Ventral view of the thorax.

Heterocerous diatrisic, average size of 20.0 ± 0.45 mm long and 5.0 ± 0.54 mm wide (*n* = 4) (Fig. 6 A). Heterochromatic pigmentation, with four main colors: white, black (predominant), gray (8/5B) in the Munsell® table and in the RGB code (# E0E0E0), and brown (10YR 7/8) in the Munsell® table and in the RGB code (# E0E0E0) (Fig. 6 AG). Head with chaetosemata absent, small in comparison to the thoracic and abdominal tagmae. Golden pectinate antennae along their entire length, including the scape; 36 pectens (Fig. 6 E). Frontal furrow; white frons, vertex, and occiput. Piliform scales prolonged on the frons, vertex, and occiput, white, darker. Immediately behind the epicranium, on the back of the thorax, there is an oblong area of black color, a fundamental characteristic in the identification of *S. ampliophilobia* (Fig. 6 B). Labial palps covered with white scales, with the apex naked and of a golden color. Missing proboscis. Ocellus absent. Large compound eyes (Fig. 6 D). Ventrally, between the cephalic and the thoracic tagma, there are dark brown piliform scales (Fig. 6 C and G). Thorax dorsally black. Coxa, trochanter, femur and half of the tibia covered with long white piliform scales; black tarsus, white sutures. Simple, long epiphysis, covered with white scales, black tip. Heteroneural wings, covered with white, brown and black scales. Very long piliform scales on the upper wing base, starting from the tegula and the humeral plate onwards. Long piliform scales below the insertion of the front wing. Anterior wing with highly developed CuP, *M bifida*, ScP and tubular RA, developed, A1 and AA3 + 4 + AP1 + 2 extended. Corda and M are in a cell. Termen present, with a staining pattern of two opposing spots, a dark black and the other faded gray (disc mark). Cordal and jugal margins of a brown color, with gray striations. Rear wing with ScP + RA developed, AP1 + 2 and AA3 + 4 extended, A1 and CuP highly developed. Corda and M in cell. Short fimbriae on both wings. Abdomen covered with short white scales (Fig. 6 F). The sexual dimorphism is only appreciable in size, where females are approximately 30% larger than males. The colors of the borer worm imago could be cryptic of lichens and epiphytic species that grow on guava trees in PV and HRS.

Some species of the genus *Simplicivalva* have already been reported for Central and South America, such as *S. charmion*, *S. corita, S. eberti, S. interrogationis,* and *S. striolata* in Brazil*, S. marmorata* in French Guiana, *S. morgani* in Jamaica, *S. philobia* in Panama and *S. poecilosema* in Bolivia (Davis et al., 2008). However, there are no reports of this genus in Colombia up to date, being the current study the first report of a species of the *Simplicivalva* genus for the country. In addition, with this report, the distribution of *S. ampliophilobia* has now expanded to Colombia.

### 3.6 Lifecycle

Twenty pupae were collected in two sampling days, 60 larvae in another visit, and 4 adults in the field, meanwhile 120 eggs and five adults were obtained in the laboratory. Light colored traps yielded adverse results in the collection of imagos after 120 hours effort/man of collection. Nevertheless, the Wilkinson-type UV-light trap after 48 effort/man hours managed to capture two imagos and reduced the number of Coleoptera and Lepidoptera individuals captured by other means (Table 4). With the plastic bottle trap, two newly emerged adults were captured after waiting for six months; the PVC trap produced no captures. No eggs were found in the field after reviewing 1,248 g of soil and bark, wounds, epiphytes and parasites associated with guava trees. Due to this, we presume that the egg is hidden using cryptic coloration against the background or by its coating, either with an agent or with secretion.

**Table 4:** Individuals of different insect orders captured, including individuals of *S. ampliophilobia* after 120 effort/man hours.

Taxon	Green light	White light	Blue light	Red light	Yellow light	Intersection∗	Wilkinson
*S. ampliophilobia*	0	0	0	0	0	0	2
Lepidoptera	0	27	59	43	50	37	8
Coleoptera	0	67	41	57	45	50	21
Neuroptera	0	6	0	0	5	15	2
Diptera	5	89	22	30	77	11	10
Hymenoptera	0	3	0	0	8	0	1

∗ Captured with an insect net.

**Fig. 7:**
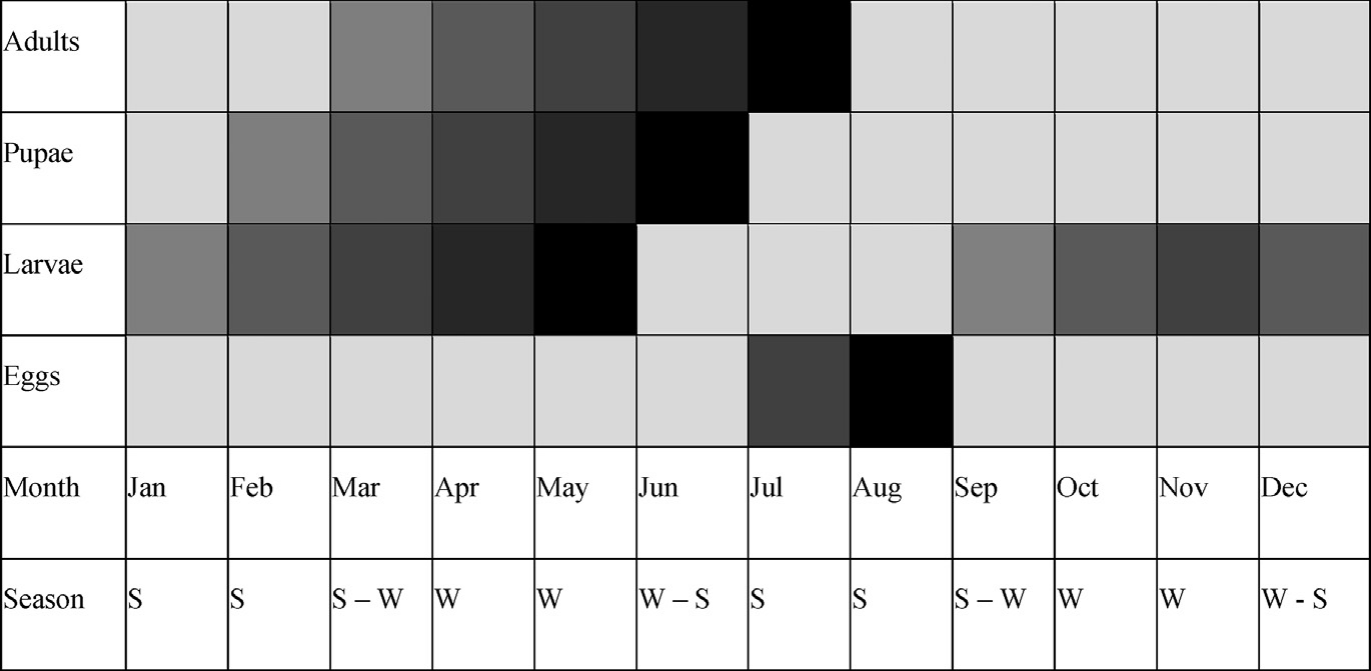
Prevalence of *S. ampliophilobia* stages over time. Darker colors indicate a higher frequency of collection per insect stage, and therefore, a higher presence. In the second month after the appearance of the guava borer worm, there is usually a higher frequency of the pest. Egg data are derived from the presence of the other stages of *S. amphilobia* in the field. S: Summer; W: Winter.

There were 61 consecutive records of population densities per randomized collection, from July 4, 2013, to December 24, 2014, and the lifecycle of *S. ampliophilobia* in the field was established as taking approximately 330–360 days (11–12 months), with one generation per year (univoltine). The egg stage usually takes 15–30 days (0.5–1 month); the larval stage takes 270 days (9 months), the prepupa stage takes 15 days (0.5 month); the pupa takes 30 days (1 month) and the adult lives 15–30 days (0.5–1 month) (Fig. 7).

**Fig. 8:**
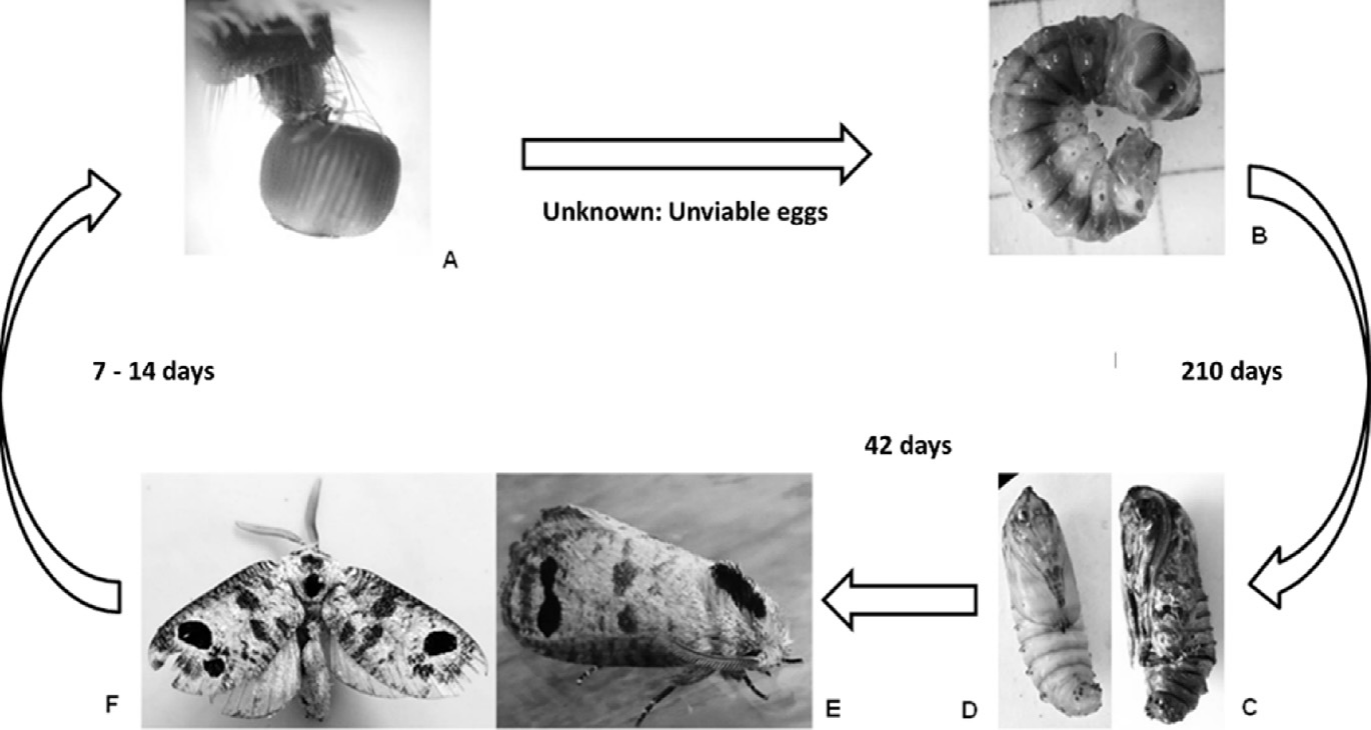
Lifecycle of *S. ampliophilobia* in the laboratory. A. Egg emerging from the ovipositor. B. Last instar larva. C. Male pupa *ad portas* to completing the stadium. D. Female pupa at the beginning of the stadium. E. Profile of the female imago. F. Male imago.

In the laboratory, with an average relative humidity of 55.6% ± 11.58% and an average temperature of 25.4 °C ± 4.93 °C, the lifecycle duration of the different stages was: egg, unknown (no fertile eggs were obtained); larva, 7 to 8 instars, 210 days; pupa, 42 days; imago, 7–14 days. Nonetheless, the lifecycle was shorter in the laboratory than in the field, lasting from 259 to 266 days (Fig. 8), adjusting to the lifecycles of the Cossidae that lasts, in areas with a winter season, up to 3 years (Huawein et al., 1993).

There was a reduction in the size and mass of the adults obtained in the laboratory compared to those collected in the field (Fig. 6 F). This situation reflects the inefficiency in the coverage of the high nutritional requirements of xylophagous larvae as well as the difficulty in breeding them due to occupied niches; another explanation could be the null copula encounters. In this regard, no diet exceeded 10% survival in the laboratory after 50 days of evaluation, so we considered there was a failure in establishing a mean to supplement the natural diet. Diet C obtained 8.3%, as did diets Zp, and CL, and with the diet Hg there was no survival. The main drawback of the diets was the consistency of the preparation, which showed a high tendency to lose water and condensation. However, the levels of methyl p-hydroxybenzoate in alcohol, known as nipagin, prevented the degradation of diets under these conditions. Nipagin is added from 1 g in 325 cm^3^ of medium (Shorey and Hale, 1965), from 1.07 g in 402 cm^3^ of medium (Greene et al., 1976), and from 1.25 g in 212 cm^3^ of medium in more recent works (ICRISAT diet, in Romero et al., 2012). Its value seems to be discretionary according to the researcher, but from the work of Oballe and Vargas Osuna (1998), where they obtained axenic means with 1.1 g in 800 cm^3^ of medium, its value was 1.375 g (1.4 g) in a liter. On the other hand, Claro and Ruiz (2007) established that the primary holidic component of a diet should be over 2% of the diet composition, a condition fulfilled by the diets used.

### 3.7 Damage description

#### 3.7.1 Incidence

We monitored 124 farms in 54 villages located in 9 municipalities of two departments, corresponding to 113.68 ha cultivated with guava, 80% of a red regional variety and 20% of a white variety. On average, 0.93 ± 0.131 ha were cultivated with guava per farm (*n* = 122). After 518 days, an average incidence of 7.51 ± 1.69 of infested trees was found (i.e. 916 trees of 4.970 trees assessed) over 40.74 ± 5.52 trees observed per farm (4,970 trees out of 18,954 trees in total). Ninety-four percent (94%) of the farms reported the presence of *S. ampliophilobia* (Table 5). Another drilling pest was found occupying the same niche of *S. ampliophilobia* in western Boyacá, with 38% presence per farm. Everything indicates that this species is a larva of the genus *Plagionotus* Mulsant (1842) (Coleoptera: Cerambycidae). This shows that the niche in *Psidium guajava* is a resource available for xylophagous species, including various orders. Added to this, *Myrtus communis* has been reported as an alternate host found in the field. This is consistent with reports made for families, genera, and species of pests where they parasitize a wide spectrum of trees of different families, a reason that exacerbates the need for consensual control actions that imply a commitment of all the chain actors, especially producers and collectors, without which the control efforts are insufficient. This fact highlights the need for pest control, but not its extermination, as a vacant niche is quickly occupied by opportunistic species, or worse, by foreign species without predators in the region (Gullan and Cranston, 2005).

**Table 5:** Presence of *S. ampliophilobia* in plots sampled in Santander and Boyacá during the second half of 2013.

Dept.	Zone	Municip.	Vda.	Farms∗	1	2	3	4	5	6	7	8	9	10	11	12	13	14	15	16	17	18	19	Total	%
Santander	Depression of the Suárez River	Guavatá	5	14	+	+	+	+	+	-	+	+	+	+	+	+	+	+						13	93
		Vélez	4	18	+	+	+	+	+	+	+	+	+	+	+	+	+	+	+	+	+	+		18	100
		Barbosa	5	10	+	+	+	+	-	+	+	+	+	+										9	90
Boyacá		Moniquirá	9	16	+	+	+	-	+	+	+	+	+	+	+	+	+	+	+	+				15	94
Santander		Pte. Nal.	10	19	+	+	+	+	+	+	+	+	+	+	+	+	+	+	+	+	+	+	+	19	100
		J. María	6	14	+	+	+	+	+	+	+	+	+	+	+	+	+	+						14	100
		S. Benito	5	13	+	+	+	+	-	+	+	+	-	+	+	+	+							11	85
Boyacá	Western Boyacá	Tununguá∗	5	13	+	-	+	+	+	-	-	-	-	+	+	+	+							8	62
		Briceño∗	5	7	-	-	-	+	-	-	-													1	14
2	2	9	54	124																				108	94

∗ Excluded from the analysis because another pest was reported in the same niche. +: Present (found); -: Absent (Not found); Dept.: Department; Municip.: Municipality; Vda.: Vereda (County).

#### 3.7.2 Severity

**Fig. 9:**
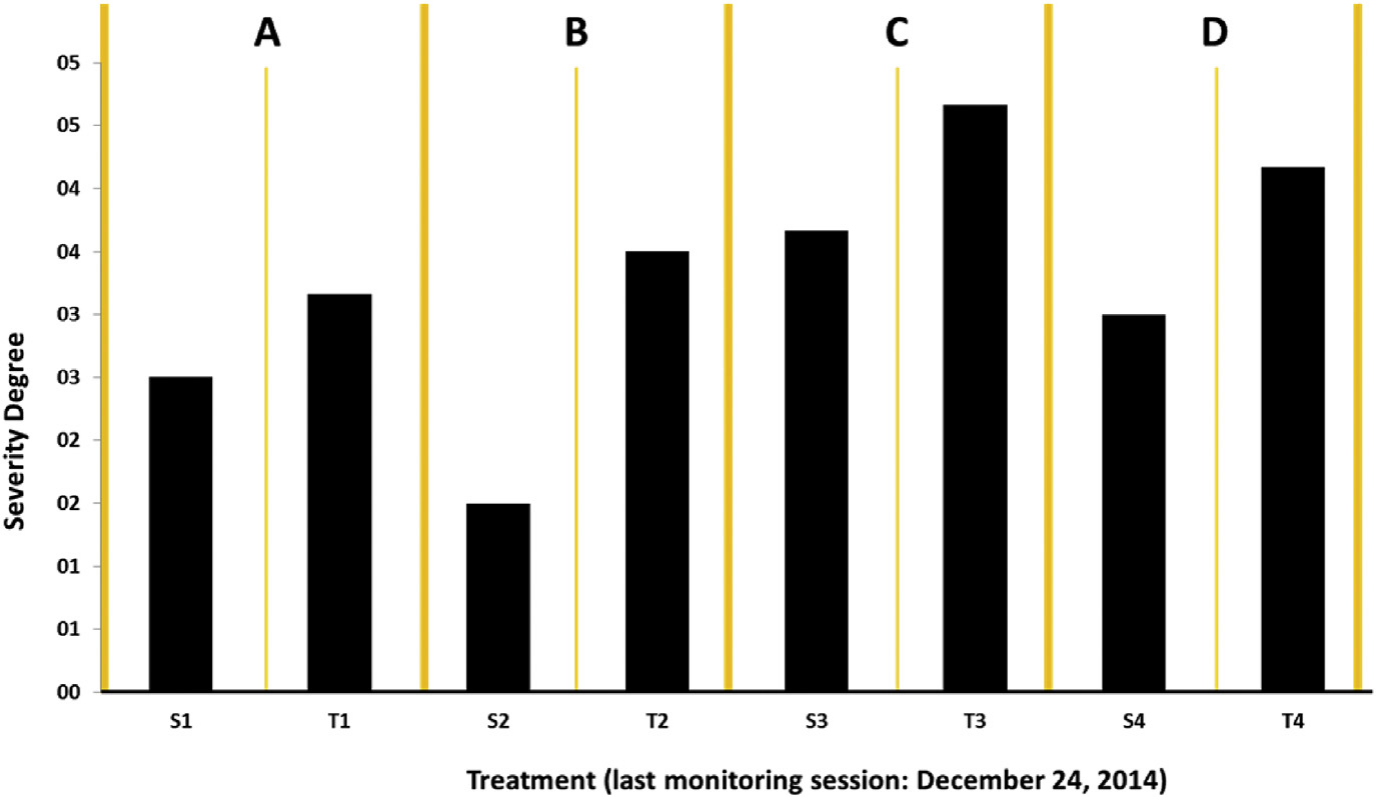
Average severity recorded for the borer worm under study in the trees evaluated after 61 follow-up weeks. A. Farm 1 Moniquirá (Finca Villa Mónica, Rosendo Pineda). B. Farm 2 Moniquirá (El Triunfo, Carlos Motta). C. Farm 3 Vélez (El Paraíso, Rosalba Ramírez). D. Farm 4 Vélez (El Pomorosal, Carlos Vargas). S: healthy trees. T: trees affected by the borer. Damage scale: 0 (Absent), 1 (Very mild), 2 (Mild), 3 (Moderate), 4 (Severe), 5 (Very severe).

4,392 entries were recorded in 61 follow-up weeks where 72 trees were evaluated. On average, the severity registered after 518 days of follow-up was moderate (3 on the severity scale) for the pest under study. Nevertheless, in all four evaluation sites, the mode was severe (4 on the severity scale). For sites 1 and 2 of Moniquirá a mode of 3, almost 4 was registered; for sites 3 and 4 of Vélez a mode of 5 was observed (Fig. 9).

**Fig. 10:**
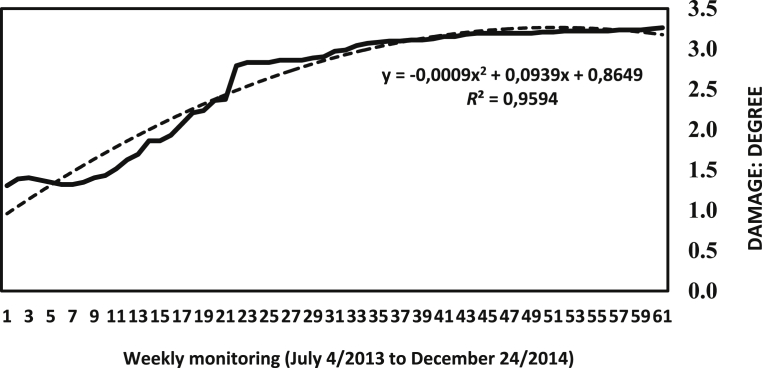
The tendency of accumulated damage over a year of records in four study locations. Damage scale: 0 (Absent), 1 (Very mild), 2 (Mild), 3 (Moderate), 4 (Severe), 5 (Very severe).

Compared with the data of the immediately preceding year, a sustained tendency to increase the damage caused by the pest is clear (Fig. 10); this shows an establishment of the populations of each species in the guava crops monitored.

Reddening of leaves towards the apex, clumped chlorosis at the edge of the leaves, rickets, epinastia, gradual loss of vigor, loss of apical dominance, the emergence of branches below the secondary and tertiary branches affected, were evidenced. Up to nine simultaneous wounds made by *S. ampliophilobia* were found. However, no death of the tree was recorded. Recovery of trees and change of the external cortex every six months was observed. This fact allows trees to get rid of pests and parasites that thrive on them, especially when they are in their early development stages.

**Fig. 11:**
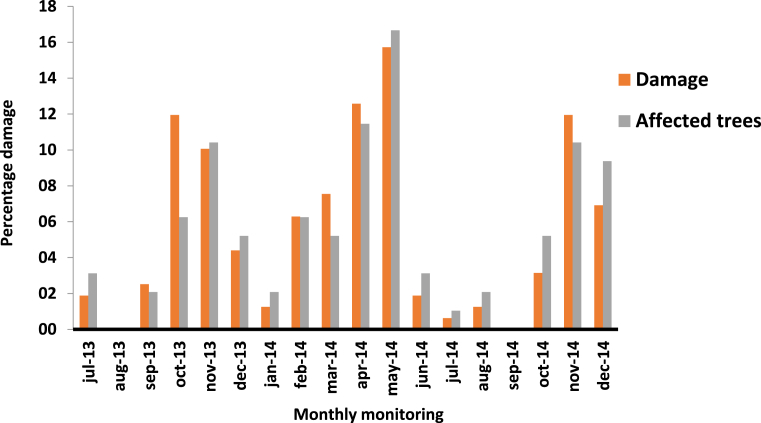
Changes in the damage caused by the larval phase of *S. ampliophilobia* over a year of monitoring in the *provincia de Vélez*, Santander, and in the HRS between Santander and Boyacá.

Regarding the evolution of damage over time, two peaks were recorded between October–November and April–May, with only one damage continuing during the year, which is accentuated towards the end of May falling abruptly with two months of almost complete inactivity between August and September (Fig. 11). These data were matched with the bimodal rain cycles of the equatorial tropics (Fig. 7). This reinforces that *S. ampliophilobia* has one generation per year with a prolonged lifecycle and little or no overlapping stages.

#### 3.7.3 Damage

The exact oviposition site of *S. ampliophilobia* females is unknown, as well as the behavior of the newly hatched larvae. However, after a month and a half to two months inside the tree without showing activity (there is no hole or excreta), accumulation of small excreta at the base of the tree can be observed. In this stage, the larva has only one gallery and does not venture much towards the center of the tree. The period between hatching and the first symptoms of activity corresponds to a phase of high voracity and accumulation of excreta. Once established in the gallery, the larva will remain there for 8–10 months, piercing a gallery of 80 cm on average, in a vertical direction, which is operculated with silk that covers excreta. Larvae seek proximity to the tree marrow and pierce in a direction parallel to this. In young trees, they consume it. It is not common for the larvae to leave the gallery, but some have been observed showing this behavior. When this happens, the larva is covered with a silk dome that protects the entire body. Under this protection, it consumes the wood horizontally until reaching the xylem vessels. Once it reaches these, it augers vertically near the medulla. A thin perimeter line remains from the silk dome, and the rest adheres to the last excreta that will be eliminated through the orifice (Fig. 1 B). The size of the excreta is an indicative of the size of the larvae. The drilling runs towards the apex of the branches or the stem, hence, it is not uncommon to find larvae boring towards the root. A larva can interrupt its vertical drilling, perform a small horizontal passage, and continue consuming tissue vertically. This means that simple networks of galleries are formed and that the same larva has two to four holes for excretion and respiration (Pulido, 2014). Once the larva has obtained a critical mass, it stops feeding and moves near the gallery with the largest hole. There it makes a silk reticule, and then wraps itself in a yellow silk cocoon and remains on its head as a prepupa (Fig. 5 G, H). After 42 days, the pupa breaks the cocoon and the reticulum and anchors to the exit orifice. From there the adult emerges, leaving the pupa exuvia. The great majority of the holes operculated by larvae were registered below 70 cm in height. Holes containing pupae predominates in secondary branches ca. 1.20 m in height. The galleries are 10 °C below the environmental temperature and 15 % points above the relative humidity value. Plant tissue removals are fatal in trees younger than five years that have small stem diameters; on the other hand, larger trees with thick stems withstand larvae attacks. We have found up to 20 simultaneous galleries made by a single individual. When dealing with the elimination of the conduction tissues of the guava plant, damages associated with transport occur. There are drastic tissue removals where only the outer cortex remains; this occurs in affected tertiary branches.

### 3.8 Management alternatives

#### 3.8.1 Cultural method

A total of 1,368 records were obtained from 342 trees during four monitoring months. The incidence percentages varied from 3.2% for a technified system up to 100% in a silvopastoral system. Likewise, within the same system of the same location, there were variations, e.g. from 16 to 21% in a technified system and from 46 to 74% in the silvopastoral system. The technified system showed a reduction in the average incidence of 50.83% compared to the silvopastoral system. This fact is attributed mainly to the use of cultural practices that are recommendable for adequate crop management. To avoid the presence of the pest, it is necessary to carry out practices such as weeding at the base of the trees to eliminate plants that can be reservoirs for the biological stages of the insect. In addition, pruning, especially formation pruning favors the entry of light that ensures an adverse environment for the borer, with the subsequent increase in temperature of the stem and branches.

#### 3.8.2 Parasitoids

A parasitoid of the genus *Apanteles* sp. was identified. On average, of 10 larvae brought from the field, six showed koinobiont superparasitism from this species.

#### 3.8.3 Entomopathogenic fungi

The entomopathogenic fungi of larvae found in this study belonged to the genus *Paecylomyces* sp. (Samson, 1974), possibly the nematophagous fungi *P. lilacinus*, *Lecanicillium lecanii* (R. Zare and W. Gams, 2001), cf. *Metarhizium anisopliae* (Metchnikoff) Sorokin, *Hansfordia* sp., and *Gliocladium* sp. (Corda, 1840).

Regarding chemical agents used in the region to control the guava borer worm, the main control substance is the use of pyrethroids in 90% of the cases, and 10% of organophosphates (Malathion® 50). The use of an insecticide called Success® 48, based on Spinosad, has increased and its use is currently widespread throughout the region.

## 4 Conclusions

The guava borer was identified as *Simplicivalva ampliophilobia* (Davis et al., 2008). Under natural conditions, the lifecycle in the field lasted 330–360 days (11–12 months), with one generation per year (univoltine). Under laboratory conditions, it lasted 259–266 days. *Simplicivalva ampliophilobia* is currently disseminated in the PV and the HRS, with incidences of 94% and moderate to severe crop affectations where it is established. Another drilling pest was found occupying the same niche of *S. ampliophilobia* in western Boyacá. This shows that the niche in *P. guajava* is a resource available for xylophagous species. Crop technification, together with the parasitoid *Apanteles* sp., and the fungi *Lecanicillium lecanii* and *Metarhizium anisopliae* are important management alternatives for the new pest in guava called the borer worm, identified in this work.

## Declarations

### Author contribution statement

Victor Camilo Pulido-Blanco: Conceived and designed the experiments; Performed the experiments; Analyzed and interpreted the data; Contributed reagents, materials, analysis tools or data; Wrote the paper.

Orlando Ildefonso Insuasty-Burbano: Conceived and designed the experiments; Performed the experiments; Analyzed and interpreted the data; Contributed reagents, materials, analysis tools or data.

Zaida Xiomara Sarmiento-Naizaque: Performed the experiments; Analyzed and interpreted the data; Contributed reagents, materials, analysis tools or data.

Julio Ramirez Duran: Conceived and designed the experiments; Contributed reagents, materials, analysis tools or data.

### Funding statement

This work was supported by Ministerio de Agricultura y Desarrollo Rural (MADR), Colombia; and Corporación Colombiana de Investigación Agropecuaria, AGROSAVIA, through the Public Funds Agreement 1828 of 2013.

### Competing interest statement

The authors declare no conflict of interest.

### Additional information

No additional information is available for this paper.
